# New treatment paradigm with systemic therapy in intermediate-stage hepatocellular carcinoma

**DOI:** 10.1007/s10147-022-02166-0

**Published:** 2022-05-08

**Authors:** Masatoshi Kudo

**Affiliations:** Department of Gastroenterology and Hepatology, Faculty of Medicine, Kindai University, 377-2, Ohno-Higashi, Osaka-Sayama, Osaka, 589-8511 Japan

**Keywords:** Hepatocellular carcinoma, Systemic therapy, Molecular targeted therapy, Immune checkpoint inhibitors, Immune microenvironment

## Abstract

Since the approval of sorafenib for the treatment of unresectable hepatocellular carcinoma in 2007 (in 2009 in Japan), five more regimens have been approved: lenvatinib, and atezolizumab plus bevacizumab for first-line treatment, and regorafenib, cabozantinib, and ramucirumab for second-line treatment, which are currently available for clinical use. The positive results of durvalumab, a programmed cell death ligand 1 antibody, plus tremelimumab, an anti-cytotoxic T-lymphocyte-associated protein 4 antibody, were also presented at the 2022 American Society Clinical Oncology Gastrointestinal Cancers Symposium as superior to sorafenib in prolonging the overall survival; this combination is expected to be approved by the end of 2022. These systemic therapies are changing the treatment paradigm not only for advanced hepatocellular carcinoma but also for intermediate-stage hepatocellular carcinoma. This review focuses on the role of systemic therapy in intermediate-stage hepatocellular carcinoma.

## Introduction

**Fig. 1:**
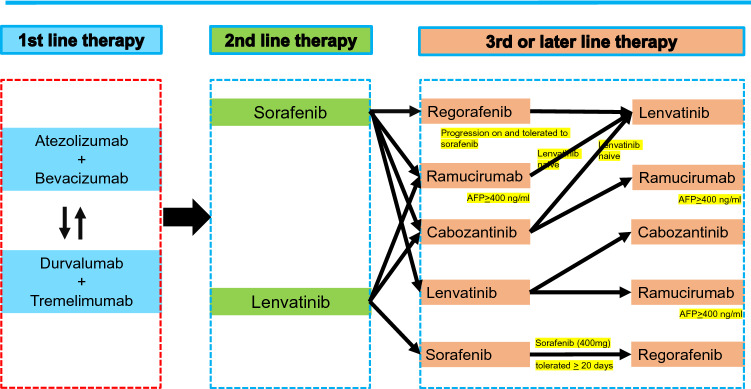
Possible sequential systemic therapy for hepatocellular carcinoma. Both atezolizumab + bevacizumab and durvalumab + tremelimumab will be the first-line systemic therapy. When one regimen is selected, another first line regimen will be selected as second-line regimen since in that way substantial triple regimen (anti-PD-L1 + anti-CTLA-4 + anti-VEGF) will be possible. ( Modified from ref# 50)

In Japan, the approval of sorafenib in 2009 marked a new era in the treatment of hepatocellular carcinoma (HCC)[1, 2]. However, many phase III clinical trials had failed to show survival benefit as first- and second-line treatments for advanced HCC [3–21](Table 1). Subsequently, several drugs were eventually approved for use as HCC treatment every year since 2017 [22–27], such as regorafenib in 2017 [24] lenvatinib in 2018 [22], ramucirumab in 2019 [26], and the atezolizumab plus bevacizumab combination in 2020 [25]. Currently, three regimens (sorafenib, lenvatinib, atezolizumab plus bevacizumab) are used as first-line treatment, and another three regimens (regorafenib, ramucirumab, cabozantinib) are used as second-line treatment. In addition, positive results for the combination of the programmed cell death ligand 1 (PD-L1) antibody durvalumab plus the anti-cytotoxic T-lymphocyte-associated protein 4 (CTLA-4) antibody tremelimumab showing overall survival (OS) benefit to sorafenib and the non-inferiority of durvalumab to sorafenib were presented at 2022 American Society Clinical Oncology Gastrointestinal Cancers Symposium (ASCO-GI 2022) [28], and this combination therapy is expected to be approved by the end of 2022. In addition, positive results of KEYNOTE-394 conducted in Asia was presented at ASCO-GI [29]. Also, interium analysis of COSMIC-312 was presented at ESMO-Asia, but trial is still ongoing [30]. Currently, the biggest challenge is to determine “in what order and to what patients these drugs should be administered” [31–37] (Fig. 1). In addition, clinical trials of immunotherapy are currently underway not only for the advanced stage but also for the early and intermediate stages, and the future development of HCC drug therapy is also attracting much attention [27].

**Table 1:**
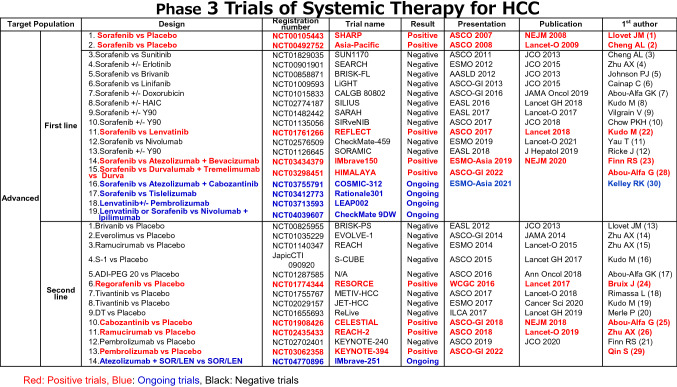
Phase 3 Trials of Systemic Therapy for HCC

**Table 2:** Definition of TACE failure/reflactoriness and TACE unsuitability (cited from ref # [42, 49, 59])

TACE failure/reflactoriness	TACE unsuitability
(1) Intrahepatic lesion	TACE-unsuitability is defined as each one of the following 3 clinical conditions that prevent a survival benefit from TACE or conditions that TACE is even harmful:
i Two or more consecutive insufficient responses of the treated tumor (viable lesion >50%) even after changing the chemotherapeutic agents and/or reanalysis of the feeding artery seen on response evaluation CT/MRI at 1–3 months after having adequately performed selective TACE	(i) Unlikely to respond to TACE: Confluent multinodular type, massive or infiltrative type, simple nodular type with extranodular growth, poorly differentiated type, intrahepatic multiple disseminated nodules, or sarcomatous changes after TACE
ii Two or more consecutive progressions in the liver (tumor number increases as compared to tumor number before the previous TACE procedure) even after having changed the chemotherapeutic agents and/or reanalysis of the feeding artery seen on response evaluation CT/MRI at 1–3 months after having adequately performed selective TACE	(ii) Likely to develop TACE failure/refractoriness: up-to-7 criteria out nodules
(2) Continuous elevation of tumor markers immediately after TACE even though slight transient decrease is observed	(iii) Likely to become Child-Pugh B or C after TACE: up-to-7 criteria out nodules (especially, biolobar multifocal nodules), mALBI grade 2b
(3) Appearance of vascular invasion	
(4) Appearance of extrahepatic spread

**Table 3:**
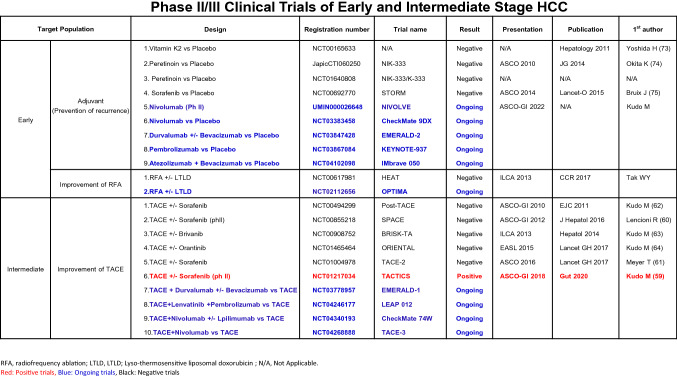
Phase II/III Clinical Trials of Early and Intermediate Stage HCC

## Role of systemic therapy in intermediate stage HCC

### Concept of TACE refractoriness

Currently, the most drastic paradigm change is the treatment strategy for intermediate-stage HCC. Intermediate-stage HCC is defined as the presence of multiple HCC nodules based on the AASLD and EASL guidelines [38, 39], and the only recommended treatment used to be TACE. In the 2017 edition of the Japan Society of Hepatology’s Clinical Practice Guidelines for Hepatocellular Carcinoma, the recommended treatment for 4 or more multiple HCCs or large HCCs of larger than 3 cm includes resection, hepatic arterial infusion chemotherapy, and molecular targeted therapy in addition to TACE [40]. In particular, the concept of “TACE refractoriness” was initially proposed in Japan in 2011 [41] and then updated in 2014 [42]. Since then, the concept of TACE refractoriness was quickly implemented in other countries worldwide [43, 44]. In Taiwan, sorafenib was initially approved for use in advanced HCC alone; given that the concept of TACE refractoriness was specified in Japan’s “Consensus-based Clinical Practice Guidelines for the Treatment of HCC” [41, 42], changes were made in Taiwan’s insurance system[45]. Using these criteria for implementing the concept of TACE refractoriness, two retrospective clinical studies showed that “patients who switched to sorafenib as soon as TACE being ineffective “showed longer survival than “patients who continuously repeat TACE after it is not effective”[46, 47]. In the OPTIMIS study [48], a global non-interventional prospective study conducted to validate the results of the retrospective clinical study, clearly showed that switching to molecular targeted therapy at the time of TACE refractoriness was more effective in prolonging the survival [48]. As a result, this “concept of TACE refractoriness and early switch to molecular targeted therapy at that point” has become a global consensus.

### Concept of TACE unsuitability

Recently, the concept of “TACE unsuitability” has been proposed in Asia and Japan [49, 50]. This concept refers to the following three conditions: (1) the condition of being susceptible to TACE refractoriness, (2)  the condition in which the liver function can easily deteriorate to Child–Pugh class B after receiving TACE, and (3) the condition of resistance to TACE (Table 2). The “Consensus Statement and Recommendation on the treatment strategy for intermediate-stage HCC” was published by the APPLE Expert Panel [49] and the Expert Panel of the Japan Society of Hepatology (HCC Treatment Manual) [50]. A patient who exceeded the up-to-seven criteria is susceptible to TACE refractoriness or to become to Child–Pugh class B. In such cases, lenvatinib is expected to (1) induce tumor necrosis and achieve downstaging, (2) inhibit recurrence by suppressing the release of hypoxia-inducing VEGF as a result of TACE, and (3) normalize the tumor vessels to enhance the effect of TACE when administered before TACE. In fact, LEN-TACE sequential therapy prolongs the prognosis of patients exceeding the up-to-seven criteria, compared with TACE [51]. This LEN-TACE sequential therapy is gradually becoming a common approach for patients in Japan who have TACE unsuitability [52]. Lenvatinib is also effective in patients with TACE-resistant conditions such as confluent multinodule type HCC, and poorly differentiated HCC [53, 54]; TACE is more beneficial in these populations when lenvatinib is introduced before TACE [55]. In fact, the evidence of TACE efficacy was established by conducting a meta-analysis of 6 randomized controlled trials comparing TACE and no therapy [56]. Meanwhile, no comparative trials have performed whether TACE or upfront systemic therapy is superior. In that sense, upfront systemic therapy prior to TACE for TACE-unsuitable patients may be a choice of treatment to achieve complete response (pathological CR) while preserving liver function[52].

The latest AASLD treatment algorithm by the AASLD Expert panel has been revised to include systemic therapy as a treatment option in addition to TACE as the recommended initial treatment for HCC patients with high tumor burden[57]. This means that the concept that was initially proposed in Japan is gradually applied overseas.

### SORA-TACE sequential therapy

The administration of molecular targeted agents with VEGF inhibitory activity prior to TACE may normalize the tumor blood vessels and increase the microvascular density, tumor interstitial pressure, and vascular permeability, thereby enhancing the efficacy of TACE through improved drug delivery [58]. This is the rationale for combining TACE with molecular targeted agents with VEGF inhibitor. To date, TACE has been used along with different molecular targeted agents in 6 clinical trials, all of which showed negative results except for the TACTICS trial [59]. The primary endpoint was PFS/TTP in TACTICS trial [59], SPACE trial [60], TACE-2 trial [61], and Post-TACE trial [62], but only the TACTICS trial showed positive results, with a PFS HR of 0.59 (95% CI: 0.41–0.78) [59]. The PFS of the BRISK-TA [63] and ORIENTAL [64] trials was also significantly favorable (PFS HR: 0.61, 95% CI: 0.74–0.99 for BRISK-TA; PFS HR: 0.86, 95% CI: 0.74–0.99 for ORIENTAL). However, the primary endpoint of the BRISK-TA trial and the ORIENTAL trial was OS, thus indicating that the trials failed to show its clinical benefit [63, 64]. In terms of OS HRs, the BRISK-TA and ORIENTAL trials, with OS as the primary endpoint, as well as SPACE, TACE-2, and Post-TACE, with OS as the secondary endpoint, showed no significant prolongation of OS as compared with patients treated with TACE alone. However, since the TACTICS trial significantly prolonged the PFS, which was the primary endpoint, the OS result was anticipated, which was the coprimary endpoint. However, the final OS data made available during the ASCO-GI 2021 showed that patients treated with a combination of TACE plus sorafenib had an OS of 36.2 months (95% CI 30.5–44.1), while those with treated with TACE alone had an OS of 30.8 months (95% CI 23.5–40.8, HR 0.86, 95% CI 0.61–1.22; *P* = 0.40); therefore, the results were considered negative [65]. The factors contributing to this negative result were as follows: (1) 156 patients set as Phase 2 trial were underpowered to meet the OS endpoint and (2) 76.3% of patients in the TACE alone group received post-treatment (50% of whom were treated with sorafenib), resulting in an extremely long post-progression survival (PPS: 17.3 months). However, considering that the OS results were negative despite the longest OS (36.2 months) and ΔOS (5.4 months) among previous combination trials of TACE and a molecular targeted agent, The results clearly showed it is no longer possible to use OS as the primary endpoint in future trials of TACE plus systemic therapy in an era with various many effective post-treatment options [65].

In any case, the results of the TACTICS trial proved that the combination of TACE and molecular targeted agents can prolong the PFS, which is the co-primary endpoint. Considering the correlation between OS HR and PFS HR in the six TACE combination trials to date, the correlation coefficient (*r*) is 0.56, clearly showing that PFS HR was poorly correlated with OS HR [65]. This result is in contrast to Llovet et al.'s plot of PFS HR and OS HR for primary and second-line agents used in patients with advanced HCC, which shows a moderate correlation coefficient of *R*=0.84 [57, 66]. In the case of combination therapy with TACE and molecular targeted agents, the impact of PPS prolongation with post-treatment is much stronger than that with first- and second-line treatments for advanced HCC, and the actual impact of PFS on OS is much weaker, which possibly led to the negative results. In addition, the regression line of the correlation of the six trials to date are somewhat smoother than those for advanced HCC, suggesting that the TACE combination trial was more strongly influenced by PPS [65]. In the future, as recently stated in the AASLD guidelines, the PFS [57]or ORR [67] could be used as surrogate endpoint for TACE combination trial since PPS has improved and OS can no longer be verified due to the effect of multiple highly effective post-treatment therapies.

The TACTICS trial also showed that (1) PFS and OS prolongation in patients exceeding up-to-seven criteria were superior to those within the up-to-seven criteria, and (2) clinically meaningful PFS and OS prolongation were observed even in patients within the up-to-seven criteria by a combination therapy of TACE and sorafenib [65].

### LEN-TACE sequential therapy

**Fig. 2:**
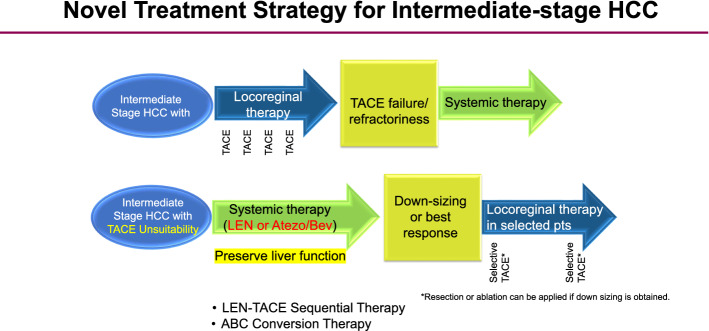
Novel treatment strategy for intermediate stage HCC. For intermediate-stage HCC unsuitable for TACE, LEN–TACE sequential therapy or ABC conversion therapy should be applied. In both cases, systemic therapy should be used upfront, and curative treatments such as resection, ablation and curative TACE should be followed to achieve a cancer-free and drug-free status

Previous studies showed that the use of lenvatinib as initial treatment may be better for improving the prognosis than use of TACE alone in patients who are unsuitable for TACE, such as those with bilobar multiple nodules [52]. In 2019, the Proof-of-Concept study showed that for cases exceeding the up-to-seven criteria, the upfront lenvatinib followed by TACE resulted in a favorable treatment effect [51]. This study included a comparison of the treatment outcomes of 37 patients who received the upfront lenvatinib in TACE-naïve patients who exceeded the up-to-seven criteria as an initial treatment for intermediate-stage HCC and 642 patients who received TACE alone. Of these, 30 patients in the upfront lenvatinib group, excluding 7 patients with observation periods of 6 months or less, were compared with 60 patients in the TACE alone group, whose characteristics were matched by propensity score matching. First, in terms of changes in liver function based on the ALBI scores, TACE caused a more irreversible deterioration in liver function compared with lenvatinib group. The PFS was also significantly longer in the lenvatinib group (16.0 months) compared with that in the TACE alone group (3.0 months) (HR: 0.19, 95% CI 0.10–0.35, *P* < 0.001). The OS was also clearly better in the lenvatinib-TACE sequential therapy (LEN-TACE sequential therapy) group, with OS of 37.9 months in the LEN-TACE sequential therapy group and 21.3 months in the TACE alone group (HR 0.48, 95% CI 0.16–0.79, *P* < 0.01). About 70% of the patients in the lenvatinib group received TACE, and four of these patients achieved a complete response and achieved cancer-free, drug-free status (including one patient who was drug free after treatment with lenvatinib alone). Thus, LEN-TACE sequential therapy showed favorable results in patients exceeding the up-to-seven criteria, which were previously extremely difficult to control with TACE alone. Therefore, lenvatinib, which provides a very high response rate, should be used as first-line treatment for intermediate-stage HCC patients exceeding the up-to-seven criteria. The extremely high response rate and preservation of liver function associated with LEN-TACE sequential therapy compared with TACE alone were the main reasons why the LEN-TACE sequential group showed good OS. The response rate of lenvatinib was 40.6% in the REFLECT study, while the response rate was 61.3% in the Japanese patients with intermediate-stage HCC [68]. The response rate in this Proof-of-concept study was 73.3%, which is extremely high. The reason for this high response rate is that many TACE-naïve patients have an ALBI grade 1 liver function, and have fewer adverse events (AEs) and lower rates of dose reduction, and discontinuation; this findings suggest that dose intensity of lenvatinib can be maintained for long time [69]. The high response rate is thought to be due to the following reasons: (1) it induces tumor shrinkage and necrosis, (2) when additional TACE is performed later, superselective TACE has a curative effects and thus preserves the liver function; (3) when lenvatinib is administered as initial treatment, it suppresses the release of hypoxia-inducible VEGF and other cytokines, thereby inhibiting recurrent metastasis; and (4) by normalizing the tumor blood vessels with lenvatinib, the permeability of blood vessels is reduced and the tumor interstitial pressure is lowered, which makes it easier for lipiodol-containing anticancer drugs to spread more evenly in the entire tumor, thereby enhancing the embolization effect and achieving pathological CR. Consequently, the administration of lenvatinib prior to TACE therapy is a theoretically effective treatment for intermediate-stage HCC patients exceeding the up-to-seven criteria, and is now becoming a common treatment strategy for intermediate-stage HCC with a high tumor burden (Fig. 2). The paradigm of the therapeutic strategy for HCC is currently undergoing a major change, as there is little evidence showing the demerits of administering lenvatinib prior to TACE, in patients with a high tumor burden.

**Fig. 3:**
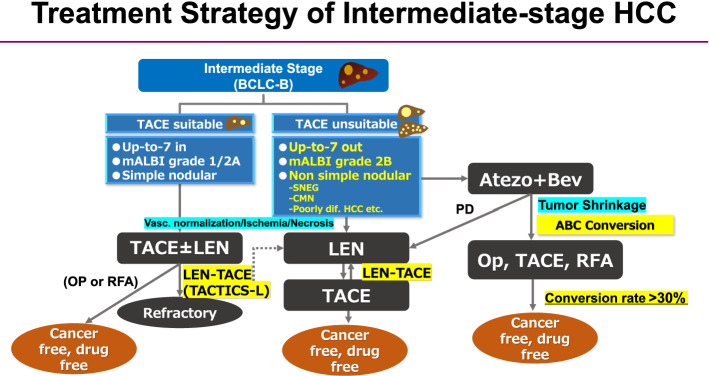
Treatment strategy of intermediate-stage HCC. *SNEG* simple nodular type with extra growth, *CMN* confluent multinodular type, *LEN-TACE* Lenvatinib-TACE sequential therapy, ABC conversion therapy, atezolizumab plus bevacizumab followed by curative conversion therapy

For bilobar multiple nodules, the administration of lenvatinib as initial treatment is undoubtedly the most ideal way to achieve a high response rate without deteriorating the liver function. In addition, for large HCCs (5 cm or larger), the amount of lipiodol in a single cTACE is often insufficient, requiring divided sessions of TACE. In addition, DEB-TACE is sometimes performed for patients with large HCC; however, there is often high risk of residual cancer at the tumor margins, or that the VEGF, FGF, angiopoietin-2 will be released, inducing rapid recurrence and metastasis. In both bilobar multiple and large HCC cases, if lenvatinib is administered in advance to normalize the tumor blood vessels, suppress the increase in VEGF expression after TACE, and reduce the residual tumor volume prior to the performance of selective TACE, a very good therapeutic effect can be obtained, and the liver function can be preserved. As a result, lenvatinib is a reasonable treatment for TACE-unsuitable patients who are likely to become refractory to TACE, such as those exceeding the up-to-seven criteria; it has the potential to become the first-line treatment for intermediate-stage HCC patients with a high tumor burden, patients with TACE-resistant HCCs, or patients with poor liver function of modified ALBI grade 2b [70]. Patients with poorly differentiated HCC showed better response to lenvatinib [53, 54]. Lenvatinib is also effective in patients with confluent multinodular type HCC and simple nodular type with extranodular growth. LEN-TACE sequential therapy may be a reasonable and effective treatment strategy for not only patients exceeding the up-to-seven criteria, but also for those with TACE resistant HCCs or with a modified ALBI grade 2b [52, 71](Figs. 2 and 3).

### ABC conversion therapy

The atezo+bev combination therapy is a combination regimen that was approved in 2020 based on the positive IMbrave150 trial [23]. The ORR in the intermediate stage was 44% under RECIST 1.1, indicating an extremely high response rate [72].

Of the 102 patients treated with the atezo+bev in a total of 3 institution, 74 have been followed up for more than 12 weeks. Of the 74 patients with a Child–Pugh grade A who were treated with first-line atezo+bev therapy, 24 (32.4%) achieved curative conversion therapy such as resection, ablation or curative TACE (Atezo/Bev followed by curative conversion: ABC conversion), and all were cancer free and drug free. Among the 24 patients, 6 underwent resections, 5 underwent radiofrequency ablation, and 12 underwent curative TACE [71]. As a result, an extremely high curative conversion rate of 32.4% was achieved. Of the 4 patients with PET-positive intermediate-stage HCC, all of 4 had curative conversion (resection 2, ablation 1, TACE plus ablation 1) and achieved cancer free and drug free status (100%) [71]. This finding indicates that atezo+bev, unlike molecular targeted agents, has markedly reduced the tumor size in responders, and it has a strong tumor shrinkage effect even in patients with very aggressive PET-positive HCC such as confluent multinodular type HCC and the poorly differentiated HCC. In some patients who underwent resection, ablation, or curative TACE, it is possible to achieve pathological CR and become drug free (ABC conversion therapy) (Figs. 2 and 3).

In general, it is common practice in the field of oncology to start a systemic therapy and continue the same regimen as long as the patient showed good response. This concept is equally true for advanced HCC as well. However, in case of intermediate-stage HCC without vascular invasion or extrahepatic spread, when tumor reduction is achieved, ablation and curative TACE are very effective measures in addition to resection to achieve pathological CR [71]; therefore, continuing the systemic therapy is not recommended in case of intermediate-stage HCC. Even if deep tumor shrinkage is achieved with atezo+bev therapy, the possibility of curative conversion at the PR status should be considered. The prognosis in patients who achieve curative conversion is extremely good; thus, systemic treatment for intermediate-stage HCC should be a completely different concept from the sequential therapy using multiple effective drugs in advanced HCC.

**Fig. 4:**
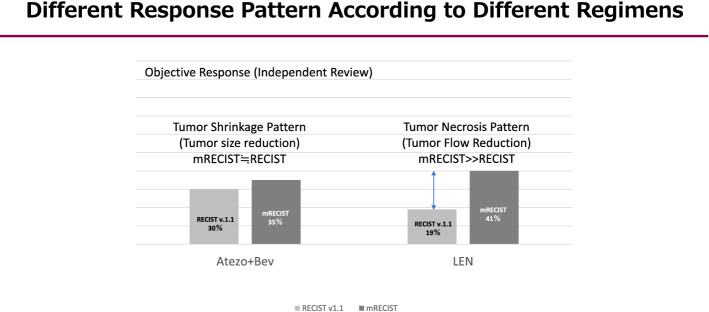
Different response pattern according to different regimens. Atezo + Bev usually achieves tumor shrinkage, whereas lenvatinib (LEN) achieves tumor necrosis through tumor arterial flow reduction. ( Modified from ref # 22 and #23)

As mentioned earlier, intermediate-stage HCC patients treated with atezo+bev showed a response rate of 44% [72]. This result indicates that one out of every two patient has the potential to achieve curative conversions. In other words, the response of intermediate-stage HCC patients to atezo+bev therapy is extremely high; if a deep response is achieved, the patient should not continue the drug until PD occurs, but should immediately switch to a curative treatment without hesitation. This is because, as is the case with lenvatinib, it is almost impossible to achieve pathological CR with systemic therapy alone, such as atezo+bev; even if it appears to be CR according to the mRECIST, viable cancer often remains after resection. Hence, curative conversion should therefore be performed. Bevacizumab should not be administered for at least 6 weeks in patients who underwent resection and at least 3 weeks in those who underwent ablation or TACE to provide a such duration between treatments in order to avoid bleeding risk during the procedure. In any case, curative conversion can be achieved in >30% of patients with intermediate-stage (ABC conversion therapy) (Figs. 2 and 3). Incidentally, a difference was observed in the 2 treatment strategies, LEN-TACE sequential therapy and ABC conversion therapy. Atezo+bev was used to achieve tumor shrinkage, while lenvatinib was used to reduce the tumor blood flow and necrosis (Fig. 4).

Therefore, there are several treatment option in treatment strategy of intermediate-stage HCC (Fig. 3).

## Future perspective

**Fig. 5:**
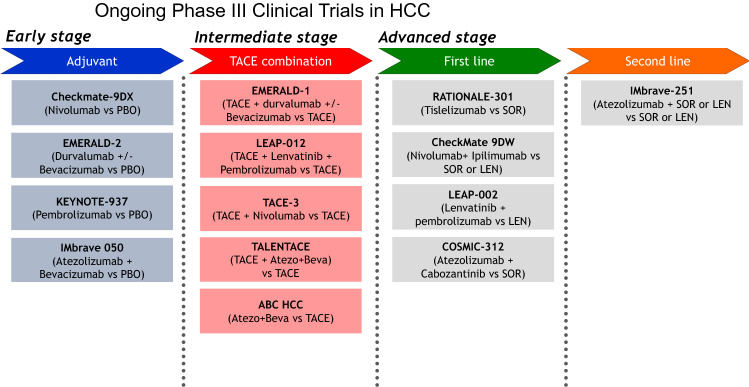
Ongoing phase III clinical trials on hepatocellular carcinoma. A number of ongoing trials are being conducted in patients with early-, intermediate-, and advanced-stage hepatocellular carcinoma using immune checkpoint inhibitors alone or in combination with tyrosine kinase inhibitors, anti-VEGF antibody or anti-CTLA-4 antibody. *PBO* placebo, *LEN* lenvatinib, *SOR* sorafenib

As shown in Fig. 5, ongoing phase III clinical trials on the efficacy of anti-PD-1/PD-L1 antibodies alone or in combination with anti-VEGF/TKIs or anti-CTLA-4 antibodies are conducted not only in patients with advanced-stage HCC but also in those with intermediate- and early-stage HCC. The results of immunotherapy adjuvant trials are highly anticipated, especially since all previous clinical trials in adjuvant setting have failed [73–75](Table 3). The positive results for advanced HCC highly suggest that clinical trials on immunotherapy (+anti-VEGF antibody/TKI) for intermediate- and early-stage HCC will be successful. If this happens, OS in patients with early and intermediate-stage HCC will be dramatically improved.

## Data Availability

N/A.

## Abbreviations

PD-L1: Programmed cell death ligand 1
CTLA-4: Cytotoxic T-lymphocyte-associated protein 4
ASCO: American society of clinical oncology
HCC: Hepatocellular carcinoma
OS: Overall survival
CR: Complete response
AE: Adverse event
